# Clinical differences between elderly and non-elderly chronic subdural hematoma patients

**DOI:** 10.3389/fneur.2026.1763619

**Published:** 2026-03-03

**Authors:** Yongxiang Yang, Dongbo Zou, Yuan Ma, Kexia Fan, Xiansong Zhu, Yunxing Li, Wanlin Liang, Jie Wang, Jingmin Cheng, Tao Yang

**Affiliations:** 1 Department of Neurosurgery, The General Hospital of Western Theater Command, Chengdu, China; 2 College of Medicine, Southwest Jiaotong University, Chengdu, China; 3 The Affiliated Hospital of Southwest Medical University, Luzhou, China; 4 School of Clinical Medicine, Chengdu Medical College, Chengdu, China

**Keywords:** chronic subdural hematoma, clinical characteristics, clinical outcome, elderly, non-elderly

## Abstract

**Objective:**

Chronic subdural hematoma (CSDH) is a common disease in elderly population, which can also be seen in young and middle-aged individuals. In order to understand the clinical features of this disease deeply, we conducted this retrospective study to clarify the clinical differences between elderly and non-elderly CSDH patients.

**Methods:**

According to the inclusion and exclusion criteria, 268 elderly CSDH patients and 87 non-elderly CSDH patients were recruited from January 2015 to December 2024 in our cohort. The collected data and compared parameters including baseline clinical features and radiological outcomes of hematoma within 24 h of hospital admission, as well as the treatment method and clinical outcome of elderly and non-elderly CSDH patients.

**Results:**

Compared to non-elderly CSDH patients, female was more frequent, the traumatic events were less common, and the history of taking anticoagulants and antiplatelet drugs were more common in elderly CSDH patients (all *P* < 0.05). Moreover, the hematoma was thicker and larger, the usage of atorvastatin was more common, the recurrence rate was higher, and the mean hospital stay was longer in elderly CSDH patients than in non-elderly CSDH patients (all *P* < 0.05).

**Conclusion:**

Elderly CSDH patients were less likely to have the traumatic events and more likely to have the history of taking anticoagulants/antiplatelet drugs, who were also owing the higher rate of using atorvastatin and hematoma recurrence, compared to non-elderly CSDH patients.

## 1 Introduction

Chronic subdural hematoma (CSDH) is a commonly encountered neurosurgical disease which is considered to be a distinct type of intracranial hemorrhage (1). CSDH is featured by a collection of blood and altered blood products in the intracranial subdural space that liquefies over time (2). It is generally assumed that CSDH is most usually present in the elderly individuals with a history of head trauma and/or using antithrombotic medication (3). While often incidentally, CSDH can also be seen in non-elderly (young/middle-aged) population. CSDH patients in different age group may have unique clinical features, as age is an essential factor in the course of disease. Until now, whether there are clinical differences between elderly and non-elderly CSDH patients is unclear.

CSDH is a gradually formed collection of blood and related products within a dual membrane capsule in the subdural potential space, whose underlying pathological mechanisms are involving a sustained process of chronic inflammation, exudation, angiogenesis and recurrent bleeding initiated by brain trauma (4–6). Age is a multifactorial risk factor for the development of CSDH. For one thing, the brain volume will decrease as the age growing, which will result in altered tension mechanics along the dura and create a potential space for the blood collection to form (7). For another, multiple age-dependent cellular and molecular alterations have been founded in the immune and angiogenic pathways in the pathological mechanisms of CSDH (6). In addition, the prevalent use of anticoagulation/antiplatelet medication, and the increased risk of ground level fall and other age related brain trauma in the aged people facilitate the formation of CSDH as well (7). Taking together, age is an important factor affecting the pathophysiology of CSDH and deserves more in-depth study.

At present, many researchers have illustrated the clinical characteristics, surgical treatment, pharmacologic interventions and outcomes of elderly patients with CSDH (4–6). However, few studies have investigated the surgical or pharmacologic treatments and functional outcomes of the non-elderly (young/middle-aged) patients with CSDH. The clinical features, treatment strategies and functional outcomes in non-elderly CSDH patients may differ from those in elderly ones. Accordingly, the perioperative management and surgical strategies should be adjusted to the age. Based on current situations, we have conducted this retrospective study to clarify the differences of clinical features, surgical treatments, pharmacologic interventions and functional outcomes between the elderly and non-elderly CSDH patients.

## 2 Materials and methods

### 2.1 Setting

This retrospective study was conducted, and the clinical data of elderly and non-elderly CSDH patients was collected at The General Hospital of Western Theater Command in Chengdu, China. Professional neurosurgeons and nurses in the department of neurosurgery in this hospital were responsible for carrying out treatments and care plans for CSDH patients. Ethics committee of the faculty of The General Hospital of Western Theater Command gave permission for this research. All the studying processes had been implemented in accordance with the approved guidelines.

### 2.2 Patients

Elderly (Age≥60years) and non-elderly (Age < 60years) CSDH patients were recruited from the inpatient service of The General Hospital of Western Theater Command in Chengdu, China. The detailed admission and hospitalization information of all patients with CSDH from January 2015 to December 2024 were carefully noted down. The inclusion criteria including: (1) Age≥18 years; (2) The discharge diagnosis was CSDH. Patients with following situations were excluded: (1) The admission and hospitalization data was incomplete. (2) Presence of extracranial injury (such as orthopedic/cardiac/chest/abdominal/pelvis traumatic injury and so on). (3) Pre-existing severe cardiac diseases (such as myocardial ischemia/infarction, heart failure). (4) Combined with liver/renal/lung failure, hematological disease, infection disease, malignancy, pregnancy.

### 2.3 Clinical care of patients with CSDH

Once patients arrived at the department of neurosurgery in our hospital, standard treatments and management were carried out immediately. All the CSDH patients received comprehensive neurological evaluation and underwent cranial CT scan subsequently. And, repeat CT scan was conducted when patients showed the indication of clinical deterioration or the sign of intracranial pressure elevation. Moreover, other routine clinical examinations including chest X-Ray, abdomen ultrasound, electrocardiogram and laboratory tests including hematology, urine and feces analysis, lipid and coagulation profile, multiorgan (cardiac, liver, renal) function analysis were conducted within 12 h after patients were hospitalized. Once the patient was diagnosed as CSDH, appropriate treatment including surgical hematoma evacuation through burr-hole craniotomy, the medication therapy including dexamethasone/atorvastatin, and conservative observation would be conducted according to the Expert Consensus on Drug Treatment of CSDH in 2020 Edition.

### 2.4 Data collection

According to the existing knowledge and latest literature report, all parameters that might be different between elderly and non-elderly CSDH patients were recorded and analyzed in this study. Specifically, the collected data including baseline clinical features and radiological outcomes of hematoma within 24 h of hospital admission, as well as the treatment method and clinical outcome of elderly and non-elderly CSDH patients. Baseline clinical features involved demography, main symptoms, initial GCS, mode of trauma and medical history. Radiological outcomes of hematoma included hematoma location, maximum thickness, midline shift and hematoma volume based on the CT/MRI presentation. Treatment methods consisted of borehole drainage, atorvastatin and dexamethasone. And, clinical outcomes were evaluated by neurological function, hospital stays, recurrence and total costs at 3 months after hospital discharge. According to the treatment and follow-up plan, patients who underwent surgical treatment will receive head CT re-examinations on the first and seventh days post operation, once a month in the following 3 months at outpatient follow-ups, or in a timely manner when patients develop new clinical symptoms. If the patients are unable to come to the hospital for outpatient follow-up, two professional neurosurgeons will conduct a telephone follow-up for them. The data of patients with complete clinical and imaging follow-up information will be subjected to subsequent statistical analysis, otherwise, they will be regarded as lost to follow-up. Recurrence was defined as the reappearance of hematoma and the indication for ipsilateral reoperation within 3 months after the initial surgery.

### 2.5 Definition of CT imaging

Based on the head CT images of CSDH patients, two professional neurosurgeons described and analyzed the location, density, separation, thickness and volume of the hematoma, and the midline deviation distance of the brain. The location of hematoma includes left, right and bilateral, as well as the brain regions such as the frontal lobe, temporal lobe and parietal lobe. The density of hematoma reflects different CT signals depending on its contents. Low density presents as uniform black or dark gray, indicating well-liquefied old hemorrhage. Isodensity is similar to the gray scale of brain parenchyma, making diagnosis rather difficult, which relies on indirect signs such as disappearance of sulci, ventricular compression, or midline structural shift for inference. High density is manifested as white, indicating the presence of fresh and active bleeding. Mixed density is most common, characterized by high-density fibrin deposits or fresh bleeding foci floating in a low-density background, forming a “liquid-liquid plane” or a mixed appearance, suggesting that the hematoma is in a process of repeated bleeding and staged coagulation. Whether there is a septum in the hematoma cavity is determined by carefully observing whether there are linear or membranous high-density shadows inside the hematoma. The presence of a septum divides a single-luminal hematoma into a multilocular one, which is a manifestation of hematoma organization and encapsulation. Hematoma thickness refers to the maximum thickness of the hematoma measured vertically on the CT image showing the thickest layer. The degree of midline deviation of the brain refers to the distance of the midline structures of the brain (such as the sickle of the brain and the hyaline septum) shift to the opposite side. The volume of hematoma is estimated using the commonly used simple ellipsoid formula in clinical practice: Volume (mL) ≈ [long diameter (cm) × wide diameter (cm) × layer thickness (cm) × number of scan layers]/2.

### 2.6 Data analysis

Measurement data was expressed as mean values ± standard deviations (M ± SD). Differences between two groups were analyzed by Unpaired *t* test with Welch's correction. Enumeration data was analyzed by Chi-squared test. SPSS version 18.0 software (SPSS Inc., USA) was used to perform the analysis, and two-tailed *P* < 0.05 was considered statistically significant. Photoshop software (Adobe Software, Inc., USA) was used to draw the figure.

## 3 Results

### 3.1 Patients selection

**Figure 1:**
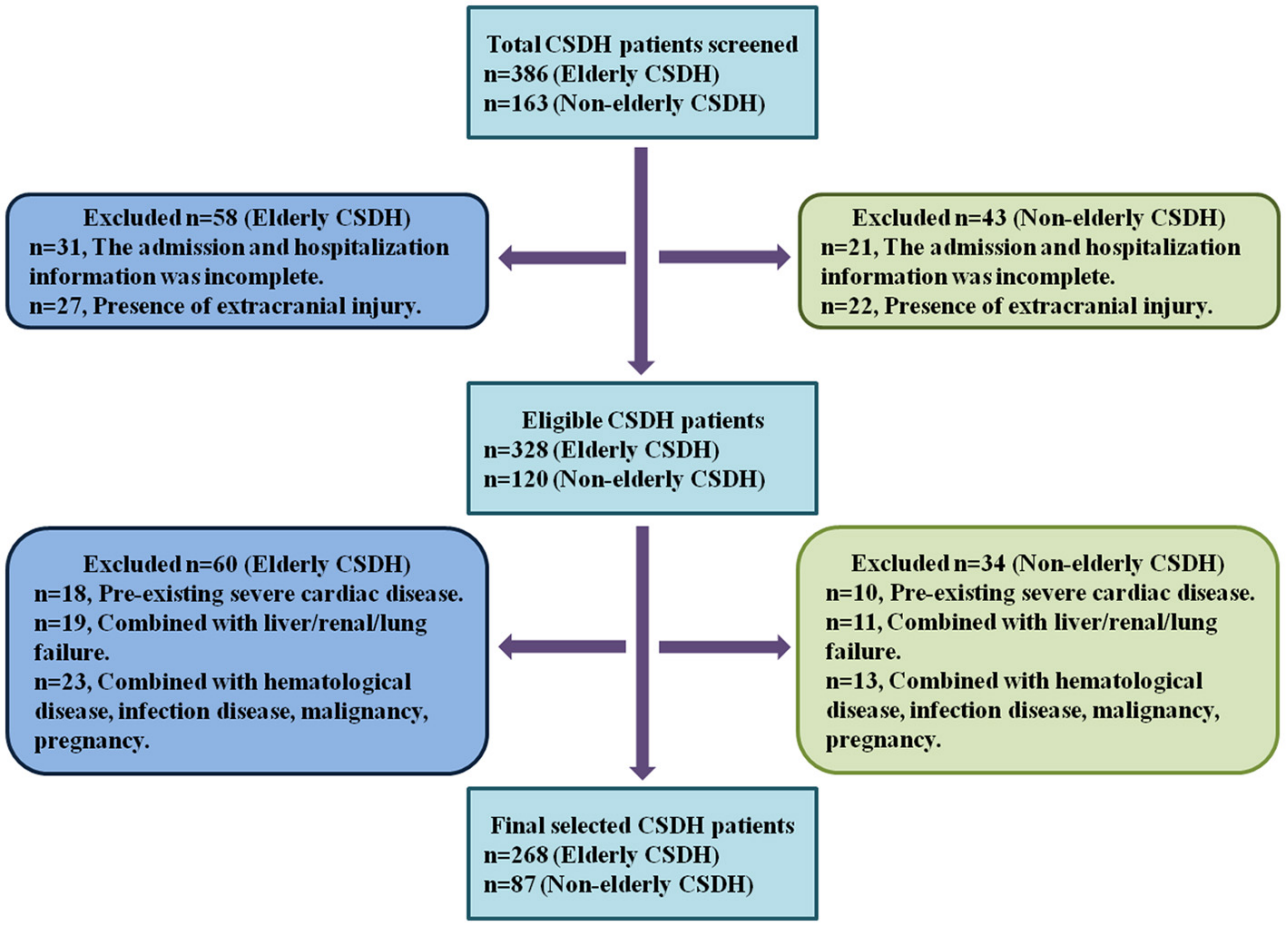
The selection flowchart of elderly and non-elderly CSDH patients.

First, all CSDH patients were recruited from the inpatient service of The General Hospital of Western Theater Command in Chengdu from January 2015 to December 2024 by using ICD-9 procedural code terminology. Then, elderly and non-elderly CSDH patients were further selected according to the inclusion and exclusion criteria as described in “Materials and Methods” section. At last, 268 elderly CSDH patients and 87 non-elderly CSDH patients were recruited in the study. The selection flowchart was illustrated in Figure 1.

### 3.2 Elderly and non-elderly CSDH patients had different baseline clinical features

Elderly and non-elderly CSDH patients had different gender distribution, as the proportion of female was higher in the former (20.9% vs. 9.2%, *P* = 0.014), but the proportion of male was higher in the latter (90.8% vs. 79.1%, *P* = 0.014). The most frequent age distribution in elderly CSDH patients was 61–80y (66.8%), and in non-elderly CSDH patients was 41–60y (81.6%). Elderly and non-elderly CSDH patients had similar severity of illness indicated by the mean GCS score, but had different main symptoms. Headache & dizziness was more frequent and limb weakness was rarer in non-elderly CSDH patients than in elderly CSDH patients (65.5% vs. 44.1% and 9.2% vs. 36.9%, *P* < 0.001). Most of non-elderly CSDH patients (63.2%) had the history of brain trauma, but most of elderly CSDH patients (59.7%) did not have the history of brain trauma (*P* < 0.001). As for the medical history, the rate of hypertension and diabetes was obviously higher (43.7% vs. 19.5%, *P* < 0.001; 16.0% vs. 6.9%, *P* = 0.032), and the taking of antiplatelet drugs was more common in elderly CSDH patients than in non-elderly CSDH patients (10.8% vs. 3.4%, *P* = 0.037). Moreover, smoking history was more frequent in non-elderly than in elderly CSDH patients (59.8% vs. 46.3%, *P* = 0.029). The detailed data was illustrated in Table 1.

**Table 1:** Baseline characteristics of elderly/non-elderly CSDH patients at admission.

**Variable**	**Elderly CSDH**	**Non-elderly CSDH**	***T*/χ^2^**	***P* value**
**Gender (** ***n*** **, %)**			6.084	**0.014** ^*^
Male	212 (79.1%)	79 (90.8%)		
Female	56 (20.9%)	8 (9.2%)		
**Age (y**, ***n*****, %)**			/	**/**
20–40	/	16 (18.4%)		
41–60	/	71 (81.6%)		
61–80	179 (66.8%)	/		
81–100	89 (33.2%)	/		
**Main symptoms (** ***n*** **, %)**			24.940	**< 0.001** ^*^
Headache & dizziness	118 (44.1%)	57 (65.5%)		
Limb weakness	99 (36.9%)	8 (9.2%)		
Epilepsy	18 (6.7%)	10 (11.5%)		
No symptoms	33 (12.3%)	12 (13.8%)		
**Initial GCS**	13.1 ± 1.6	14.4 ± 1.3	−1.231	0.289
**Brain trauma history (** ***n*** **, %)**			13.894	**< 0.001** ^*^
With	108 (40.3%)	55 (63.2%)		
Without	160 (59.7%)	32 (36.8%)		
**Medical history (** ***n*** **, %)**			
Hypertension	117 (43.7%)	17 (19.5%)	16.256	**< 0.001** ^*^
Coronary heart disease	30 (11.2%)	5 (5.7%)	2.193	0.139
Diabetes	43 (16.0%)	6 (6.9%)	4.620	**0.032** ^*^
Anticoagulant drug	4 (1.5%)	2 (2.3%)	0.257	0.612
Antiplatelet drug	29 (10.8%)	3 (3.4%)	4.353	**0.037** ^*^
Smoking history	124 (46.3%)	52 (59.8%)	4.789	**0.029** ^*^
Drinking history	121 (45.1%)	40 (46.0%)	0.018	0.893

CSDH, chronic subdural hematoma; GCS, Glasgow Coma Scale.

*P* < 0.05 represents statistically significant (marked with ^*^ and bold).

### 3.3 Elderly and non-elderly CSDH patients had different radiological presentations

As shown in Table 2, several significant differences were founded in the CT presentations of hematoma between elderly and non-elderly CSDH patients at admission. First, the most common density of hematoma in non-elderly CSDH patients was high (52.9%), followed by equal and (27.6%) and mixed (11.5%), but the most common density of hematoma in elderly CSDH patients was equal (31.7%), high (30.2%) and mixed (27.3%) (*P* = 0.001). Second, the rate of separation in hematoma was obviously higher in elderly CSDH patients than in non-elderly CSDH patients (24.3% vs. 8.0%, *P* = 0.001). Third, both the maximum thickness and hematoma volume was obviously higher in elderly CSDH patients than in non-elderly CSDH patients (23.6 ± 7.8mm vs. 18.5 ± 6.7mm, *P* = 0.008; 114 ± 77 vs. 83 ± 62ml, *P* = 0.035). In a word, above data showed that elderly and non-elderly CSDH patients had different radiological presentations.

**Table 2:** Radiological parameters of elderly/non-elderly CSDH patients at admission.

**Variable**	**Elderly CSDH**	**Non-elderly CSDH**	***T*/χ^2^**	***P* value**
**Hematoma location (** ***n*** **, %)**			0.159	0.924
Left side	115 (42.9%)	39 (44.8%)		
Right side	99 (36.9%)	32 (36.8%)		
Bilateral	54 (20.2%)	16 (18.4%)		
**Density of hematoma**			17.246	**0.001** ^*^
High	81 (30.2%)	46 (52.9%)		
Low	29 (10.8%)	7 (8.0%)		
Equal	85 (31.7%)	24 (27.6%)		
Mixed	73 (27.3%)	10 (11.5%)		
**Separation in hematoma**			10.671	**0.001** ^*^
With	65 (24.3%)	7 (8.0%)		
Without	203 (75.7%)	80 (92.0%)		
**Maximum thickness (mm)**	23.6 ± 7.8	18.5 ± 6.7	−2.716	**0.008** ^*^
**Midline shift (mm)**	10.9 ± 4.9	9.3 ± 4.2	−1.563	0.187
**Hematoma volume (ml)**	114 ± 77	83 ± 62	−2.166	**0.035** ^*^

CSDH, chronic subdural hematoma.

*P* < 0.05 represents statistically significant (marked with ^*^ and bold).

### 3.4 Elderly and non-elderly CSDH patients had different treatment and clinical outcome

As shown in Table 3, the rate of using atorvastatin was higher in elderly CSDH patients than in non-elderly CSDH patients (70.1% vs. 55.2%, *P* = 0.01), but the rate of borehole drainage, repeated operations and usage of dexamethasone in elderly and non-elderly CSDH patients was similar. As for the clinical outcome (Table 4), the rate of recurrence was higher (24.6% vs. 13.8%, *P* = 0.03) and the mean hospital stays was longer (13.5 ± 10.5 days vs. 10.6 ± 6.6 days, *P* = 0.017) in elderly CSDH patients than in non-elderly CSDH patients. The outcome of neurological function at discharge and the total cost was similar in elderly and non-elderly CSDH patients. These findings indicated that elderly and non-elderly CSDH patients had different treatment and clinical outcome.

**Table 3:** Treatment of elderly/non-elderly CSDH patients.

**Variable**	**Elderly CSDH**	**Non-elderly CSDH**	***T*/χ^2^**	***P***
**Borehole drainage (** ***n*** **, %)**			0.251	0.616
With	153 (57.1%)	47 (54.0%)		
Without	115 (42.9%)	40 (46.0%)		
**Repeated operations (** ***n*** **, %)**			0.893	0.345
With	17 (11.1%)	3 (6.4%)		
Without	136 (88.9%)	44 (93.6%)		
**Atorvastatin (** ***n*** **, %)**			6.611	**0.01** ^*^
With	188 (70.1%)	48 (55.2%)		
Without	80 (29.9%)	39 (44.8%)		
**Dexamethasone (** ***n*** **, %)**			3.365	0.067
With	34 (12.7%)	18 (20.7%)		
Without	234 (87.3%)	69 (79.3%)		

CSDH, chronic subdural hematoma.

*P* < 0.05 represents statistically significant (marked with ^*^ and bold).

**Table 4:** Clinical outcome of elderly/non-elderly CSDH patients at discharge.

**Variable**	**Elderly CSDH**	**Non-elderly CSDH**	***T*/χ^2^**	***P***
**Neurological function (** ***n*** **, %)**			0.773	0.68
Improvement	182 (67.9%)	63 (72.4%)		
No change	61 (22.8%)	16 (18.4%)		
Deterioration	25 (9.3%)	8 (9.2%)		
**Recurrence**			4.496	**0.03** ^*^
With	66 (24.6%)	12 (13.8%)		
Without	202 (75.4%)	75 (86.2%)		
**Hospital stays (days)**	13.5 ± 10.5	10.6 ± 6.6	−2.391	**0.017** ^*^
**Total costs (wan yuan)**	2.8 ± 2.7	2.5 ± 2.4	−0.890	0.374

CSDH, chronic subdural hematoma.

*P* < 0.05 represents statistically significant (marked with ^*^ and bold).

## 4 Discussion

We clarified the clinical differences between elderly and non-elderly CSDH patients via conducting this retrospective study. Our major findings were as follows: (1) Compared to elderly CSDH patients, male was more frequent, limb weakness was rarer, the history of brain trauma was more common, and the use of anticoagulants/antiplatelet drugs was rarer in non-elderly CSDH patients. (2) Compared to non-elderly CSDH patients, the rate of separation in hematoma, the maximum thickness and the total volume of the hematoma was obviously higher in elderly CSDH patients. (3) Compared to non-elderly CSDH patients, the rate of using atorvastatin was higher, the rate of recurrence was higher, and the mean hospital stay was longer in elderly CSDH patients. These findings are helpful to demonstrate the clinical features, the surgical or pharmacologic treatments and functional outcomes of CSDH patients, especially for the non-elderly ones.

As is well-known, CSDH is a commonly encountered neurologic disorder that is increasingly prevalent in the elderly people. Recent studies have reported that the most common onset age of CSDH was 50 years old in the 1970 s, 60 years old in the 1980 s, 70 years old in the 1990 s and 80 years old in the 2010 s, which is gradually shifting toward a higher age (8, 9). One study in Japan indicated that the highest percentage of CSDH patients were 80s (36.8%), and patients over 70 years accounted for 78.2% (10). Nowadays, most of the researchers have spared their efforts to explore the clinical characteristics, surgical treatment, pharmacologic interventions and outcomes of elderly patients with CSDH (4–6). Nevertheless, few studies have investigated the clinical aspects of the non-elderly (young/middle-aged) patients with CSDH. Therefore, we conducted this study to analyze the clinical differences between elderly and non-elderly CSDH patients, thereby providing clinical references for individualized diagnosis and treatment of CSDH patients.

The proportion of female was higher in elderly CSDH patients, and the proportion of male was higher in non-elderly CSDH patients. This founding is interesting as the typical CSDH patient is a male in his seventh decade (6). The possible explanation for male was more frequent in non- elderly CSDH patients might be the fact that those patients have a higher rate of brain trauma, which is more likely to occur among the man population. Brain trauma is an essential inducement for the onset of CSDH, which can lead to the tearing of bridging veins with subsequent bleeding, thus creating the hematoma (11). Our previous research has found that CSDH without brain trauma was more common in elder people (12), which is in line with this study indicating that most of elderly CSDH patients did not have the history of brain trauma, but most of non-elderly CSDH patients had the history of brain trauma. This means brain trauma might be a more important cause of non-elderly CSDH patients than elderly ones. Except for brain trauma, the use of anticoagulants or antiplatelet drugs might be an important cause of CSDH as well, especially for elderly patients. This research indicated that the taking of antiplatelet drugs was more common in elderly CSDH patients than in non-elderly ones. This result is in accordance with most of the current researches indicating that elderly CSDH patients have a higher rate of cardiovascular and cerebrovascular diseases requiring antiplatelet or anticoagulant medication. Anti-thrombotic drugs might be an essential risk factor for the onset of CSDH, as some researchers have demonstrated that the risk of CSDH increased to 2.45 in odds ratio (OR) for patients receiving anticoagulants or to 1.42 in OR for patients receiving antiplatelets in a case-control study (13–15). Taking together, these results indicate that brain trauma is an important inducement for non-elderly CSDH patients, while the use of anticoagulants or antiplatelet drugs is an essential inducement for elderly CSDH patients.

Computed tomography (CT) scan can display direct evidences and clinical references for the diagnosis and treatment of CSDH. Under normal circumstances, non-contrast head CT provide imaging information about the density, morphology, structure, thickness and volume of hematoma. CT results of this research demonstrate that the most common density of hematoma in non-elderly CSDH patients was high, but in elderly CSDH patients was equal. Hematoma density relative to brain parenchyma on a CT image can reflect the length of bleeding time, a high density indicates recent bleeding, while a low density indicates forward bleeding (16). Non-elderly CSDH patients usually show high-density CT signals because their clinical symptoms appear in the early stage of bleeding, as a result of the plump brain tissue and small intracranial buffer space of these people. However, elderly CSDH patients often present mixed density CT signals because their clinical symptoms only appear after the space-occupying effects become obvious after repeated bleeding, due to the atrophy of brain tissue and large intracranial buffer space of these people. Apart from the density, internal architecture such as the separation in hematoma is different between elderly and non-elderly CSDH patients as well. The rate of separation in hematoma was obviously higher in elderly CSDH patients than in non-elderly CSDH patients. Some researchers found a higher recurrence risk in laminar and separated hematoma than in other hematomas (16). Moreover, the density and structure of hematoma have been employed by many researchers to classify CSDH, thereby providing clinical evidences for the formulation of treatment decisions and the prediction of hematoma recurrence. Nomura et al. have categorized hematomas into 5 types according to the density: high, isodense, low, mixed, and layering (17). And, Nakaguchi et al. (18) have classified hematomas into 4 types according to the internal architecture: homogenous, laminar, separated, and trabecular. Based on the Nakaguchi classification, some researchers have developed a new classification method, which categorized hematomas into 5 types according to the internal architecture and CT HU values : homogenous, gradation, laminar, separated, and trabecular (19). Those classifiations of CSDH had been applied to predict the recurrence of hematoma, and some researchers found the gradation-type hematoma to be a significant recurrence-related factor (19). According to the correlation between classifiation and recurrence of hematoma, the higher rate of separation in hematoma in elderly CSDH patients might indicate greater possibility of recurrence. Moreover, contrast MRI can display the density and architecture of hematoma of CSDH as well, which not only provides information about the morphology of hematoma and the extent of distribution over the cerebral lobes, but also provide precise information about the liquid and solid components of hematoma (20). One latest study have indicated that the risk of postoperative recurrence of CSDH patients with a net-like appearance on cranial MRI can be predicted by confirming structural change between the pre- and postoperative net-like appearance on MRI (21). In the future, it can be predicted that the combination of CT and MRI will construct a more precise classification system of hematomas, which may divide CSDH patients into high-recurrence-rate or low-recurrence rate groups and further be useful for the formulation of clinical treatment.

As is well-known, the main treatments for CSDH include surgical evacuation of hematoma, medication therapy, and the newly applied middle meningeal artery embolization (MMAE). For CSDH patients presenting with neurological symptoms, the first-line treatment remains to be burr-hole/twist-drill hole evacuation of hematoma (20). In our research, more than half of elderly and non-elderly CSDH patients have undergone drilling and drainage surgery, and there is no significant difference in percentage between the two groups. Though surgical evacuation can rapidly resolve mass effects and alleviate clinical symptoms, it is usually accompanied by high rates of recurrence (20% more or less) (20, 22). We find the rate of recurrence was higher in elderly CSDH patients (24.6%) than in non-elderly CSDH patients (13.8%), a difference is worth noting. The higher recurrence rate of elderly CSDH patients might be related to the alterations of brain anatomical structure and local chronic inflammatory response in the hematoma with age growing. On the one hand, brain atrophy that occurs with age growing increases the subdural space of elderly CSDH patients, thereby increasing the probability of subdural small blood vessels rupturing again and leading to the recurrence of subdural hematoma (7, 8). On the other hand, the degree of chronic inflammatory response in the local area of the hematoma is more intense and the duration is longer in elderly CSDH patients, which makes the pathological process of small vessel proliferation and bleeding induced by inflammatory response more likely to appear repeatedly, resulting in the recurrence of subdural hematoma (6). The high recurrence rate of CSDH post operation, especially in elderly patients, suggesting that surgical evacuation of hematoma alone is insufficient in addressing the chronic and recurrent nature of CSDH. At present, one effective and widely used method for treating CSDH recommended by clinical research and clinical guidelines is drug therapy, which mainly includes dexamethasone and atorvastatin. Drug therapy can be used alone to treat CSDH patients with small hematoma volume, mild symptoms, no need for surgery, and those who are unwilling or unable to tolerate surgery, it can also be combined with surgery to achieve better therapeutic effects and a lower recurrence rate of hematoma. Dexamethasone has been explored in many clinical studies for the treatment of CSDH due to its ability to inhibit inflammatory responses, as the proliferation and bleeding of small blood vessels caused by local chronic inflammatory responses are the key pathological mechanisms of the occurrence and recurrence of CSDH. However, the efficacy data of dexamethasone are mixed and overall do not support its routine use to treat CSDH patients (23). Atorvastatin also has the ability to inhibit inflammatory responses, which has shown some promise in a phase II clinical trial as it can potentially reduce the need for eventual surgery by 50% among non-surgical CSDH patients (24, 25). In our research, only a small part of elderly (12.7%) and non-elderly (20.7%) CSDH patients had used dexamethasone, but a big part of elderly (70.1%) and non-elderly (55.2%) CSDH patients had used atorvastatin. This finding suggests that elderly CSDH patients might be beneficial in facilitating the complete removal of the hematoma and reducing the recurrence rate by taking atorvastatin after surgery in clinical practice. Moreover, other newly developed drugs such as tranexamic acid (pro-coagulant) and bevacizumab (anti-angiogenic) had also being investigated, while their application in clinical practice still needs more in-depth researches to explore (25, 26). In recent years, a newly therapeutic technique named MMAE is being extensively and deeply explored in clinical research and practice. The middle meningeal artery (MMA) provides vascular supply to the subdural membranes and neo-vasculature, which is thought to be responsible for the persistence and recurrence of CSDH. Accordingly, MMAE has been hypothesized to be an effective adjunctive treatment to surgical evacuation to reduce the recurrence rate of CSDH, based on the theory that the blood supply can be restricted by endovascular embolization of MMA (20). In the beginning, MMAE was verified to be capable of reducing the recurrence rate of CSDH from more than 20% with conventional management from less than 5% (27, 28). Later on, several persuasive studies have further confirmed that MMAE is also an effective standalone treatment in promoting the resorption and resolution of CSDH without surgical evacuation, especially for those patients with smaller hematoma volumes and mild symptoms (29–32). Most recently, three key global multicenter clinical studies including EMBOLISE, MAGIC-MT and STEM have further investigated the role of adjunctive MMAE for the treatment of CSDH in addition to conventional management, which also demonstrated excellent effectiveness and safety data for MMAE (33). These findings indicate that MMAE is likely to be incorporated into the standard care for CSDH, which might be a better new treatment for elderly CSDH patients, as they have a higher incidence and poorer tolerance to surgery than non-elderly ones.

This study has the following limitations. Firstly, as a single-center retrospective study, only speculative explanations can be provided for the results such as “the potential protective effect of atorvastatin on elderly CSDH patients” and “the higher recurrence rate in elderly CSDH patients may be related to brain atrophy or enhanced inflammatory response”, and multi-center prospective studies are still needed to support and explain these research conclusions in the future. Secondly, it might be difficult to cite literatures to conduct an in-depth comparison of the differences between elderly and non-elderly CSDH patients from the aspects of pathophysiology, imaging features and clinical course, as there is almost no research on non-elderly patients with CSDH can be found. Thirdly, it might be difficult to conduct a discussion on the potential limitations or risks of MMAE such as treatment tolerance, complications and surgical risks in combination with clinical data, because only a very small number of patients with CSDH were treated by MMAE in our center. Finally, the baseline differences between elderly and non-elderly CSDH patients are inevitable and it might be difficult to conduct multivariate analyses on clinically relevant covariates, as the main objective of this study is to compare the clinical differences between these two groups of patients, rather than to analyze the clinical factors that can influence the clinical outcomes of those patients. Despite this study had the above limitations, it was the first one that conducted a comprehensive analysis of the clinical differences between elderly and non-elderly CSDH patients through a single-center, large-sample and retrospective clinical study. Moreover, the findings of this research not only have certain reference value for the conduction of large-sample and prospective clinical studies in the future, but also have some clinical application significances for the formulation of personalized treatments for patients with CSDH in different age groups.

In conclusion, this retrospective study clarified the clinical differences between elderly and non-elderly CSDH patients for the first time. First, elderly CSDH patients had a higher rate of taking anticoagulants/antiplatelet drugs, but had a lower rate of undergoing brain trauma history, compared to non-elderly CSDH patients. Second, the rate of separation in hematoma, the maximum thickness and the total volume of the hematoma was higher in elderly CSDH patients than in non-elderly CSDH patients. Third, the rate of using atorvastatin and recurrence was higher, and the hospital stay was longer in elderly CSDH patients than in non-elderly CSDH patients. These findings provided new knowledge about the clinical features, the treatment strategies and functional outcomes of elderly and non-elderly CSDH patients, which might be helpful for achieving better therapeutic effects.

## Data Availability

The raw data supporting the conclusions of this article will be made available by the authors, without undue reservation.
